# Relationship between structural brainstem and brain plasticity and lower-limb training in spinal cord injury: a longitudinal pilot study

**DOI:** 10.3389/fnhum.2015.00254

**Published:** 2015-05-06

**Authors:** Michael Villiger, Patrick Grabher, Marie-Claude Hepp-Reymond, Daniel Kiper, Armin Curt, Marc Bolliger, Sabina Hotz-Boendermaker, Spyros Kollias, Kynan Eng, Patrick Freund

**Affiliations:** ^1^Spinal Cord Injury Center Balgrist, University of ZurichZurich, Switzerland; ^2^University College Physiotherapy Thim van der LaanLandquart, Switzerland; ^3^Institute of Neuroinformatics, University of Zurich and ETH ZurichZurich, Switzerland; ^4^Institute of Neuroradiology, University Hospital Zurich, University of ZurichZurich, Switzerland; ^5^Department of Brain Repair and Rehabilitation, UCL Institute of Neurology, University College LondonLondon, UK; ^6^Wellcome Trust Centre for Neuroimaging, UCL Institute of Neurology, University College LondonLondon, UK

**Keywords:** spinal cord injury, structural plasticity, lower limb, virtual reality-augmented neurorehabilitation, tensor-based morphometry, voxel-based morphometry

## Abstract

Rehabilitative training has shown to improve significantly motor outcomes and functional walking capacity in patients with incomplete spinal cord injury (iSCI). However, whether performance improvements during rehabilitation relate to brain plasticity or whether it is based on functional adaptation of movement strategies remain uncertain. This study assessed training improvement-induced structural brain plasticity in chronic iSCI patients using longitudinal MRI. We used tensor-based morphometry (TBM) to analyze longitudinal brain volume changes associated with intensive virtual reality (VR)-augmented lower limb training in nine traumatic iSCI patients. The MRI data was acquired before and after a 4-week training period (16–20 training sessions). Before training, voxel-based morphometry (VBM) and voxel-based cortical thickness (VBCT) assessed baseline morphometric differences in nine iSCI patients compared to 14 healthy controls. The intense VR-augmented training of limb control improved significantly balance, walking speed, ambulation, and muscle strength in patients. Retention of clinical improvements was confirmed by the 3–4 months follow-up. In patients relative to controls, VBM revealed reductions of white matter volume within the brainstem and cerebellum and VBCT showed cortical thinning in the primary motor cortex. Over time, TBM revealed significant improvement-induced volume increases in the left middle temporal and occipital gyrus, left temporal pole and fusiform gyrus, both hippocampi, cerebellum, corpus callosum, and brainstem in iSCI patients. This study demonstrates structural plasticity at the cortical and brainstem level as a consequence of VR-augmented training in iSCI patients. These structural changes may serve as neuroimaging biomarkers of VR-augmented lower limb neurorehabilitation in addition to performance measures to detect improvements in rehabilitative training.

## Introduction

Complete spinal cord injury leads to permanent impairments of motor, sensory, and autonomic function. However, in the majority of cases, sensorimotor functions are partially preserved below the lesion level due to an anatomically incomplete lesion, resulting in an incomplete spinal cord injury (iSCI; Wyndaele and Wyndaele, 2006). Intensive neurorehabilitation therapy improves clinical outcome, but little is known about the relationship between training intensity and behavioral outcome, or about their neural underpinnings. The ability to relearn successfully a motor action depends on the key concepts for motor learning, i.e., repetition, performance feedback and motivation (Holden, 2005). Virtual reality (VR)-augmented neurorehabilitation interventions in patients with chronic iSCI have shown to improve motor function and neuropathic pain (e.g., Villiger et al., 2011, 2013). VR provides interactive, multimodal sensory stimuli and immediate environment feedback to motivate the patient. Furthermore, adjustments of the VR-presented motor tasks aim to prevent or reduce fatigue and demotivation (Holden, 2005; Adamovich et al., 2009; Bohil et al., 2011). However, the neurophysiological mechanisms underlying performance improvements in patients with acute and chronic iSCI are uncertain.

To date, training-dependent functional neuroplasticity in humans has been investigated using transcranial magnetic stimulation (TMS), positron emission tomography (PET) and functional magnetic resonance imaging (fMRI). Long-term motor training induces decreases in task-related activity in cortical motor areas, indicating economization processes (Walz et al., 2015) and changes in functional connectivity (Sampaio-Baptista et al., 2015). Recently, structural and functional cortical connectivity changes were associated with motor training (Taubert et al., 2011). Cortical modulation was assessed with TMS in training of upper (Pascual-Leone et al., 1995; Classen et al., 1998; Koeneke et al., 2006; Kantak et al., 2013) and lower extremities (Perez et al., 2004). Classen et al. (1998) showed that simple thumb training could change the movement direction evoked by TMS indicating reorganization of neuronal networks. Crucially, computational morphometry has shown training-dependant (i.e., juggling) dynamic volumetric changes in brain tissue in motor learning associated cortical areas in healthy controls (Draganski et al., 2004). Patients with Parkinson’s disease—trained on a balance task—showed improvements that were related to local brain volume increases in cortical and cerebellar regions involved in balance control and motor learning (Sehm et al., 2014). These increases in brain volume occurred within the first weeks of training during which the greatest improvements occurred. Thus, improvements in ambulatory function might depend on similar mechanisms in iSCI patients who have to relearn movement patterns using spared fibers that most probably were involved in different motor tasks prior to spinal cord injury (Ghosh et al., 2010).

Morphometric brain changes are conventionally assessed using voxel-based morphometry (VBM) and voxel-based cortical thickness (VBCT). Both procedures were developed for cross-sectional detection of gray and white matter volume changes between two groups (Ashburner and Friston, 2000; Hutton et al., 2008). Recently, tensor-based morphometry (TBM) was developed to assess dynamic volumetric changes over time (Ashburner and Ridgway, 2013). TBM has successfully shown its potential in longitudinal studies characterizing changes induced by SCI trauma, inflammation in multiple sclerosis, and in neurodegeneration such as Alzheimer’s and Parkinson’s diseases (Tao et al., 2009; Freund et al., 2013; Hua et al., 2013; Tessa et al., 2014).

In the present study, we applied computational morphometry (VBM, VBCT) to detect volumetric bi-directional differences between chronic iSCI patients and controls at baseline. Over time in iSCI patients, we used TBM to assess the relationship between VR motor-training induced performance improvements and local brain volume increases in brain areas responsive to motor training during a similar balance task in in healthy and diseased subjects (Taubert et al., 2010; Sehm et al., 2014).

## Materials and Methods

### Participants and Study Design

Nine iSCI outpatients (mean age 55.1 years, standard deviation (SD) 15.8, range 28–71 years, 4 females) from the University Hospital Balgrist (Zurich, Switzerland) were included in the study between August 2010 and March 2012 (Table 1). Inclusion criteria were: clinically incomplete, chronic SCI (time since injury >1 year), motor level of lesion below C4, ability to sit in a chair without assistance and support systems (e.g., securing belt), and motor functions below the level of lesion corresponding to the American Spinal Cord Injury Association Impairment Scale (AIS; Marino et al., 2003). Exclusion criteria were psychiatric or other neurological disorders, head injuries causing cognitive or visual impairment, spasticity limiting performance of lower limb movements, and medication influencing ability to attend the therapy for 45 min. Furthermore, patients with depressive symptoms (score >14 in the Beck Depression Inventory) were excluded (Hassanpour et al., 2012). Neuropathic pain was classified into at-level and below-level SCI neuropathic pain based on the most recent taxonomy (Bryce et al., 2012) and was assessed on an 11-point numeric rating scale from 0 (no pain) to 10 (worst pain imaginable).

**Table 1:** **Characteristics of patients with spinal cord injury**.

Patient	Age (years)	Gender	Etiology	Level of lesion	AIS classification	Level of pain	Years since injury
P1	70	M	ME	C8	D	-	2
P2	60	F	ME	T4	D	Below-level	2
P3	28	M	T	C6	D	-	5
P4	71	F	ME	T12	D	At-level	3
P5	61	M	T	C4	D	At- and below-level	4
P6	30	M	ME	C5	D	Below-level	3
P7	62	M	ME	T9	D	-	5
P8	67	F	T	T12	D	-	1
P9	47	F	ME	C7	D	Below-level	4

*Gender: M = male; F = female. Etiology: T = trauma; ME = medical etiology. Level of lesion: C = cervical; T = thoracic level SCI. AIS classification D: sensory-motor incomplete, with the average strength of the muscles below the level of lesion equal to or above 3 (i.e., movement over the full range of motion against gravity). Level of pain: at-level pain is defined as pain located within the dermatome and 3 dermatomes below the lesion level, and not in any lower dermatomes, unless the pain is thought to be caused by damage to the cauda equine. Below-level pain is defined as pain present more than 3 dermatomes below the lesion level, and the lesion or disease must affect the spinal cord and that the pain is believed to arise as a result of this damage*.

Fourteen healthy subjects participated in the control group (mean age 47.1 years, SD 14.4, range 25–61 years, 7 females). Their mean age was not significantly different from iSCI patients (Mann–Whitney *U* = 34.5, nc = 14, np = 9, *p* = 0.07, two-tailed). They had normal or corrected-to-normal visual acuity and no history of psychiatric or neurological disorder.

SCI and healthy participants were informed of the purpose of the study and gave written consent. The experimental protocol was in accordance with the Declaration of Helsinki and performed with the approval of the local Ethics Committee (EK-24/2009).

Patients underwent 4 weeks of intensive VR-augmented lower limb training. Before and after the training period a structural volumetric 3D MRI data set was acquired in patients. Retention of the performance improvements was assessed in a 3–4 month follow-up session. The 14 control subjects were invited to a single MRI session using the same imaging protocol as the patients to reveal any baseline differences (increases and decreases) in local brain volume between both groups. Controls did not partake in the VR-enhanced motor training.

The patients with iSCI were trained with the VR movement tasks 16–20 times during 4 weeks (4–5 × 45 min. per week). The training used a VR-augmented therapy system for lower limbs combining action observation, imagination and execution. The system and the motivating training tasks are shown and described in more detail by Villiger et al. (2011, 2013). In short, the movements of the patient’s real lower limbs are transferred in real time to the virtual lower limbs using sensory modules with accelerometers, attached to the patients’ shoes. The virtual lower limbs are presented in a first-person perspective on a large monitor (132 cm diagonal). Clinically relevant interactive games for training foot and leg movements were used for 3 × 2 min each in a sitting or standing position. These games were:

1. *Footbag*: A simple exercise in which the patient juggles a ball between the left and right foot, using dorsiflexion of the ankle (tibialis anterior contraction, approx. 70 per 2 min. per leg), an exercise to reduce foot dragging. The trajectory of the ball in the air between the left and right feet is pre-set.
2. *Hamster splash*: Hamsters run up to the patient’s toes. The patient’s task is to perform a dorsal flexion of the ankle (approx. 30 per 2 min. per leg) to launch each hamster into a swimming pool. Launching the hamsters higher (faster ankle movement) is rewarded by a higher score and more elaborate hamster movements (somersaults, swimming patterns).
3. *Star kick*: The patient performs a knee extension (approx. 24 per 2 min. per leg) by kicking a ball towards displayed stars. For every hit, the patient receives a score reward.
4. *Planet drive*: Cars are moving on a highway towards the virtual feet. The patient’s task is to avoid touching the cars by displacing the foot and legs sideways (approx. 8 per 2 min. per leg).

During each training session, patients rated their enjoyment, motivation, and attention level on an 11-point numeric rating scale from 0 (worst) to 10 (best).

### Behavioral Data

Outcomes for lower limb motor functions in patients with iSCI were assessed at four different time points: 1 month prior to baseline scan, at baseline (immediately before training), after one month of training (post-training), and 3–4 months after the last training session (follow-up). Patients were assessed on: (1) the “10 meter walking test” (10 MWT) gait speed test (self selected speed) (van Hedel et al., 2005, 2006); (2) the “Berg balance scale” (BBS) assessment of balance during functional activities (14 balance items) from 0 (no balance) to 4 (good balance) (Berg et al., 1995); (3) the “lower extremity motor score” (LEMS) from 0 (complete paralysis) to 50 (normal strength) (Marino et al., 2003); and (4) the “spinal cord independence measure” (SCIM mobility) from 0 (no mobility) to 40 (normal mobility) (Catz et al., 2007).

### Statistical Analysis of Behavioral Data

We used Stata 13 (StataCorp LP, College Station, TX)^1^ to assess changes in behavioral data. In order to estimate rates of clinical recovery (patients only) linear mixed models were used with the clinical measure (10 MWT, BBS, LEMS, SCIM) as response variable and time as predictor. All time points and all participants were included.

### MRI Data Acquisition

The structural volumetric MRI images were acquired at baseline in all participants and following training in patients. A 1.5T whole-body MRI scanner equipped with an 8-channel SENSE™ head coil (Philips Medical Systems, Eindhoven, The Netherlands) was used to acquire 3D T1-weighted (T1w) (TFE sequence) (repetition time (TR) = 20 ms, echo time (TE) = 4.6 ms, flip angle (FA) = 20°, field of view (FoV) = 220 × 220 mm^2^, 210 slices, voxel size = 0.98 × 0.98 × 0.75 mm^3^). Total scan time was 11 min. 36.4 s for each subject. All images were controlled for movement artifacts prior to processing.

### MRI Data Analysis

#### Voxel-Based Morphometry (VBM) and Voxel-Based Cortical Thickness (VBCT)

VBM (Ashburner and Friston, 2000) and VBCT (Hutton et al., 2008), implemented in SPM12 (Wellcome Trust Centre for Neuroimaging, University College London, London, UK), were applied to assess differences in white matter and gray matter volumes and cortical thickness between the iSCI patients and the healthy controls prior to training (Ashburner and Friston, 2000). In brief, for VBM the T1w anatomical images were segmented into gray matter, white matter and cerebrospinal fluid using unified segmentation (Ashburner and Friston, 2005). The gray matter and white matter segments were then transferred to MNI (Montreal Neurological Institute) space using the diffeomorphic non-linear image registration algorithm (Dartel) (Ashburner, 2007). Finally, the gray matter volumes were scaled (i.e., “modulation”) in order to preserve the local tissue volumes and smoothed using an isotropic Gaussian kernel with 6 mm full width at half maximum (FWHM). For VBCT, the segmented maps of gray matter, white matter, and cerebrospinal fluid (created in the pre-processing steps of the VBM analysis) were used to create a cortical thickness map for each subject (Hutton et al., 2008). Cortical gray matter boundaries were extracted from these maps to estimate the cortical thickness for each voxel. To increase the spatial resolution of narrow cerebrospinal fluid spaces, the input tissue segments were sub-sampled from 1 mm to 0.5 mm using trilinear interpolation. The thickness value at each voxel was then calculated as the distance between the inner and outer borders of the local gray matter (Hutton et al., 2009). VBCT maps were then warped into the MNI space and smoothed using the same FWHM Gaussian kernel with a correction to preserve (i.e., no modulation) local cortical thickness (Hutton et al., 2008).

#### Tensor-Based Morphometry (TBM)

Using inverse-consistent 3D non-linear image registration, implemented in SPM12, we longitudinally aligned the MRI volumes collected before and after training to their half-way average (Ashburner and Ridgway, 2013) to obtain Jacobian determinant maps, as well as half-way T1w average images for each subject. The half-way average images were subsequently segmented into gray matter, white matter, and cerebrospinal fluid using unified segmentation (Ashburner and Friston, 2005). The Jacobian determinant maps were transformed to the MNI space using deformations derived from Dartel. These aligned maps revealed volumetric expansion and compression for each of the subjects.

### Statistical Analysis

For the VBM/VBCT analysis, used to assess impairment related structural brain changes prior to study onset, three general linear models (GLM) were constructed for the whole brain (for gray matter, white matter, and cortical thickness), consisting of a group identifier, age, and total intracranial volume (TIV) to account for any confounding (non-specific) effects. For the cortical changes (VBM of gray matter and VBCT of cortical thickness), the sensorimotor cortex was additionally chosen as search volume to increase sensitivity (Freund et al., 2011). Two-tailed two-sample *t*-tests were used to assess volume differences in patients compared to controls at baseline.

For TBM analysis, linear regression models were constructed to identify volumetric changes that are associated with individual performance improvements (i.e., 10 MWT, LEMS, BBS, and SCIM) across the training period. Individual performances of the lower limb motor functions were assessed before and after the 4 weeks training. As the study duration was short and age has shown not to have disadvantageous effects on rehabilitation associated performance improvements (Furlan et al., 2010), we did not include age as a covariate in the TBM analysis (Freund et al., 2013). Pain reduction during training was included as covariate of no interest in TBM models to overcome possible confounds, because training with visual aspect has shown reductions in SCI neuropathic pain scores (e.g., Moseley, 2007; Villiger et al., 2013). Regression models were tested for positive correlations between volume changes and improvements in performance.

The associated *p*-values were corrected for multiple comparisons of all voxels analyzed using Gaussian random field theory. Only significant results (*p* < 0.05, Family Wise Error (FWE) corrected) are reported.

## Results

### Patient Characteristics and Motivational Factors

All patients completed the training and were classified as AIS D. Five patients suffered from neuropathic pain. The clinical characteristics are summarized in Table 1. On average, during each VR training session around 300 repetitions of ankle movements and 75 knee movements (over 5000 and 1200 per leg respectively during a month) were performed. A survey at the end of every training session indicated that patients enjoyed the VR-augmented lower limb tasks (mean 9.5, SD 0.9, range 7.7–10) and maintained a high level of motivation (mean 8.5, SD 1.2, range 6.9–10) and attention (mean 8.6, SD 1.2, range 6.4–10).

### Behavioral Data

**Figure 1:**
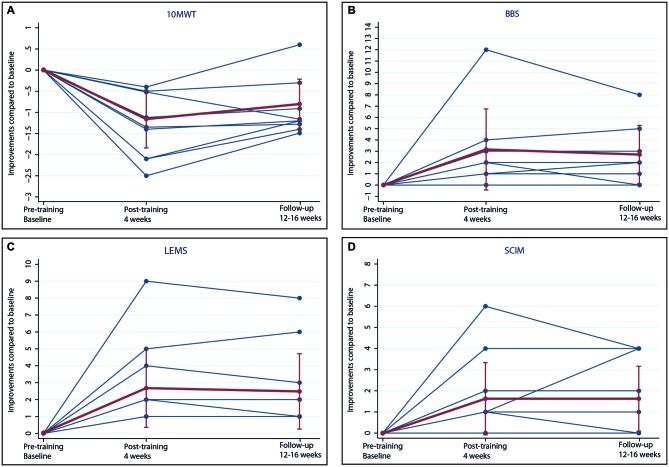
**Performance improvements**. Improvements of **(A)** 10 meter walking test (10 MWT), **(B)** Berg balance scale (BBS), **(C)** lower extremity motor score (LEMS) and **(D)** spinal cord independence measure (SCIM) in patients with incomplete spinal cord injury (iSCI) after 16–20 interactive training sessions during 4 weeks (post-training) and 12–16 weeks after training (follow-up). Individual results **(blue)** and means with standard deviations **(red)** are shown.

To control for spontaneous recovery and to assess the stability of improvement tests, no significant changes occurred between the period of time one months before training and at baseline (pre-training) (10 MWT: 0.01s, *p* = 0.999, CI −0.43–0.43; BBS: −0.15, *p* = 0.856, CI −1.73–1.44; LEMS: −0.03, *p* = 0.965, CI −1.18–1.13; SCIM: −0.25, *p* = 0.617, CI −1.22–0.72). During the entire training period, patients spent a total mean training time of 413.8 min. (SD 43.2, range 344–480). Over time, all iSCI patients exhibited training-induced improvements in performance (see Figure 1). Specifically, iSCI patients showed a significant improvement in the 10 MWT (−1.33 s, *p* < 0.001, CI −1.77 to −0.90), BBS (3.11, *p* < 0.001, CI 1.51–4.72), LEMS (3.00, *p* < 0.001, CI 1.81–4.19), and SCIM (1.89, *p* = 0.001, CI 0.81–2.97) in the post-training compared to the pre-training. The performance at follow-up was still significantly improved compared to pre-training in the 10 MWT (−0.93 s, *p* < 0.001, CI −1.37 to −0.49), BBS (2.67, *p* = 0.010, CI 0.65–4.69), LEMS (2.78, *p* = 0.002, CI 1.03–4.52), and SCIM (1.89, *p* = 0.002, CI 0.71–3.07). They did not significantly change when compared to post-training in the 10 MWT (0.41 s, *p* = 0.068, CI −0.03–0.84), BBS (−0.44, *p* = 0.587, CI −2.05–1.16), LEMS (−0.22, *p* = 0.715, CI −1.42–0.97), and SCIM (0.00, *p* = 1.000, CI −1.08–1.08).

### Cross-Sectional Structural Changes (MRI Data) Prior to Training—VBM and VBCT

**Figure 2:**
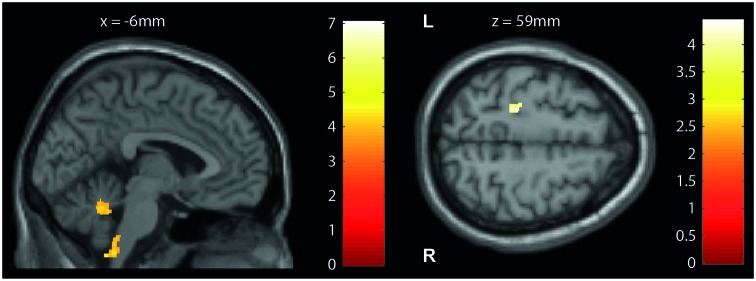
**Cross-sectional structural changes (VBM/VBCT)**. Statistical parametric maps (thresholded at *p* < 0.001 uncorrected, for illustrative purposes) showing volume reductions in patients with iSCI compared with controls at baseline. (Left) Voxel-based morphometry (VBM): cerebellum and brainstem (medulla oblongata) and (right) voxel-based cortical thickness (VBCT): left primary motor cortex. The color bars indicate the *t*-scores.

At baseline, VBM of white matter volume revealed significant reductions in the cerebellum (lobule IX, Z score 3.95, *p* < 0.001) and brainstem (medulla oblongata, Z score 4.88, *p* < 0.001) in patients compared to controls (Table 2; Figure 2, left). No alteration in VBM of gray matter volume was detected in patients compared to controls.

**Table 2:** **VBM/VBCT volume decreases (e.g., atrophy) at whole brain before training between patients and healthy controls**.

Region	*Z* score	*P* value (cluster, FWE-corrected)	Cluster extent (voxel)	MNI coordinates		
				*x* (mm)	*y* (mm)	*z* (mm)
**VBM**
Brainstem (medulla oblongata)	4.88	<0.001	300	−6	−48	−61
Cerebellum (lobule IX)	3.95	<0.001	353	−6	−51	−31
**VBCT**
Primary motor cortex*	3.63	<0.001	46	−23	−30	59

*Analysis was performed in the sensorimotor cortex to increase sensitivity (Freund et al., 2011). VBM = voxel-based morphometry; VBCT = voxel-based cortical thickness.

VBCT of cortical thickness (Table 2; Figure 2, right) revealed thinner cortical thickness in the left primary motor cortex of iSCI patients compared to controls when analyzing the sensorimotor cortex as region of interest (Z score 3.63, *p* < 0.001), but not at whole brain. No volumetric and cortical thickness increases were detected in patients compared to controls.

### Longitudinal Structural Changes (MRI Data) TBM

**Figure 3:**
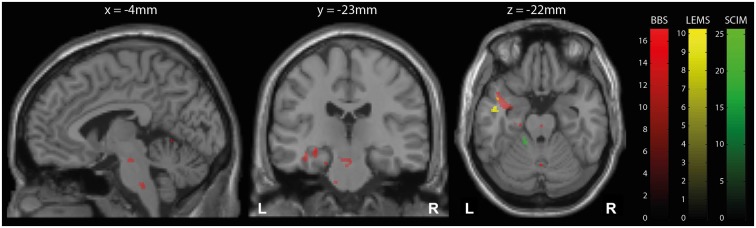
**Longitudinal structural changes (TBM)**. Overlay of statistical parametric maps (thresholded at *p* < 0.001 uncorrected, for illustrative purposes) showing correlations between volume increases measured by tensor-based morphometry (TBM) and clinical improvements in balance (BBS, red), lower extremity motor score (LEMS, yellow), and spinal cord independence measure (SCIM, green). The corresponding *t*-scores are indicated by the color bars. Correlations are shown in brainstem (left), hippocampus (middle), and left temporal gyrus and cerebellum (right).

Over the course of training, the iSCI patients’ improvements in performance correlated positively with dynamic changes in local volume in several brain regions (Table 3; Figure 3). Related to the BBS scores, the changes were located in the right cerebellum (lobule V, Z score 4.70, *p* = 0.037; lobule VI, Z score 4.58, *p* = 0.002), left middle occipital gyrus (Z score 4.28, *p* = 0.034), both hippocampi (Z score 4.27, *p* < 0.001; Z score 4.17, *p* < 0.001), the left temporal pole (Z score 4.00, *p* < 0.001), corpus callosum (Z score 4.12, *p* < 0.001), and two brainstem regions (pons, Z score 4.16, *p* < 0.001; midbrain, Z score 4.09, *p* = 0.003). For the LEMS, a positive correlation between volumetric changes and improvements were found in the left middle temporal gyrus (Z score 4.05, *p* < 0.001). The SCIM scores were positively correlated with longitudinal volume changes in the left cerebellum (lobule VI, Z score 5.17, *p* < 0.001; lobules I-IV, Z score 4.77, *p* = 0.003) and left fusiform gyrus (Z score 4.52, *p* < 0.001). No significant correlations between the behavioral and structural changes were found for the 10 MWT.

**Table 3:** **Correlations between TBM longitudinal volume increases and performance improvements in patients**.

Region	*Z* score	*P* value (cluster, FWE-corrected)	Cluster extent (voxel)	MNI coordinates		
				*x* (mm)	*y* (mm)	*z* (mm)
**BBS**
Right cerebellum (lobule V)	4.70	0.037	59	3	−57	0
Right cerebellum (lobule VI)	4.58	0.002	93	8	−68	−13
Left middle occipital gyrus	4.28	0.034	60	−39	−65	−1
Right hippocampus	4.27	<0.001	122	27	−5	−40
Left hippocampus	4.17	<0.001	159	−30	−18	−18
Brainstem (pons)	4.16	<0.001	129	−8	−32	−43
Corpus callosum	4.12	<0.001	143	−24	−6	36
Brainstem (midbrain)	4.09	0.003	89	−9	−17	−16
Left temporal pole	4.00	<0.001	833	−35	4	−36
**LEMS**
Left middle temporal gyrus	4.05	<0.001	324	−45	−2	−15	
**SCIM**
Left cerebellum (lobule VI)	5.17	<0.001	169	−15	−60	−27
Left cerebellum (lobules I-IV)	4.77	0.003	87	−14	−39	−21
Left fusiform gyrus	4.52	<0.001	265	−30	−75	−10

*TBM = tensor-based morphometry; BBS = Berg balance scale; LEMS = lower extremity motor score; SCIM = spinal cord independence measure*.

## Discussion

This study reveals specific structural brain changes associated with motor performance improvements following intensive VR-augmented lower limb training in chronic iSCI patients. As anticipated, intensive training over a period of 4 weeks resulted in improvements in ambulation (e.g., 10 MWT) and postural stability/control (e.g., BBS) (Deliagina et al., 2008). Overall independence in their daily life improved, as shown by the higher SCIM scores. The success of this intensive VR-augmented training with over 5000 ankle movements and 1200 knee movements per leg during a month of training was reflected by high ratings for enjoyment, motivation, and attention. Crucially, motor performance improvements relate to increases in local brain volume in areas relevant for locomotion. These findings allow us to address the question to what extent motor performance improvements during rehabilitation are reflected by brain plasticity.

Using longitudinal structural MRI data, it has been shown repeatedly that training improvements in healthy controls can be related to increases in cortical volume in humans (Driemeyer et al., 2008; Taubert et al., 2010; Landi et al., 2011; Sagi et al., 2012; Sehm et al., 2014) and monkeys (Quallo et al., 2009). These changes occur rather rapidly (within weeks) and the effects are stronger when a new task was learned as opposed to a refinement of an already learned task (Driemeyer et al., 2008; Quallo et al., 2009). Moreover, these structural brain changes are dynamic, demonstrating increases during the initial phase of learning and decreases at later stages (Taubert et al., 2010; Sehm et al., 2014). Only a few studies have assessed this phenomenon in neurological disorders (Burciu et al., 2013; Sehm et al., 2014). Using TBM, we demonstrate dynamic structural brain plasticity associated with motor learning in several cortical and subcortical areas in patients with chronic iSCI. These brain regions correspond with those involved in the learning of balance tasks in the healthy and diseased brain and occurred within the first weeks (Sehm et al., 2014).

In particular, the iSCI patients exhibited an expansion of gray matter volume in the temporal lobe that was associated with improvements in balance (BBS) and muscle strength (LEMS) of the lower limbs. The responsiveness of the temporal lobe is similar to that observed during upper limb training in healthy subjects who were trained over 3 months to learn a classic three-ball cascade juggling routine (Draganski et al., 2004). Furthermore, our VR training, which demanded high levels of spatial memory capacities, induced structural changes within the hippocampus. These results are in line with hippocampi volume increases associated with spatial memory and learning in a cohort of London taxi drivers memorising navigation patterns (Maguire et al., 2000). In addition, functional MRI studies have shown that the hippocampus is also involved in encoding (Schendan et al., 2003) and consolidation of motor skills (Albouy et al., 2008). The increase in gray matter volume due to VR-augmented lower limb training in the cerebellum most likely reflects improved adaptation of postural movement (Houk et al., 1996; Rabe et al., 2009; Burciu et al., 2013). An increase in volume was induced in the brainstem, but only little is known about the anatomical plasticity of key locomotor regions in this area. In a recent study with rats, Zörner et al. (2014) observed that anatomical plasticity in defined brainstem motor networks significantly contributed to functional recovery after injury of the central nervous system.

While the analysis of the MRI data can show dynamic changes over time, it cannot explain the microstructural neuronal processes underlying learning-induced brain volumetric changes (Draganski and May, 2008). Thus, the dynamic gray and white matter volume increases observed in our cohort of iSCI patients may be attributable to several factors such as synaptogenesis, astrocytic hypertrophy, neurogenesis, angiogenesis and myelin formation (for review, see Markham and Greenough, 2004).

In accordance with previous structural MRI studies, white matter volume in the cerebellum, medulla oblongata, and cortical thickness in the primary motor cortex were reduced in iSCI patients when compared to controls (Freund et al., 2011, 2013). At a microstructural level, these atrophic changes are related to axonal degeneration and demyelination (Cohen-Adad et al., 2011; Freund et al., 2012a,b). Interestingly, we observed training-induced increases in local brain volume in key areas of locomotion (i.e., brainstem and cerebellum) adjacent to areas that showed volume reductions as assessed by VBM. Future quantitative MRI studies using myelin-sensitive readouts will elucidate whether training can reverse atrophy by promoting structural integrity.

Our study had few limitations. Although we carefully selected and assessed the patients clinically, the sample size was rather small (*n* = 9, 18 MRI assessments). Therefore our study might have been insensitive to detecting small changes with week effect sizes. Moreover, the lack of a SCI control group for the VR-augmented training means that the training induced changes in trained patients might have occurred not only as part of the training but also as part of a placebo effect (e.g., secondary effect due to participation in the training study). However, the locations of the brain volume changes were strongly overlapping with those shown to be responsive during motor training in healthy subjects and neurological impaired patients and therefore we are confident that the findings relate to performance improvements rather than spontaneous recovery. Moreover, the clinical outcome measure were shown to be stable 1 months prior to training commencement indicating that the observed behavioral improvements were training-induced rather than occurring spontaneously.

## Conclusion

Overall, our present pilot study demonstrates a close link between structural changes in spatially distinct, task-specific areas of the central nervous system related to performance improvements during motor learning in patients with chronic iSCI. Novel neuroimaging measures of dynamic structural changes in response to training hold significant potential to provide complementary information regarding the intensity and specificity of functional training programs which, while applied routinely in SCI patients, may require adjustment in terms of their intensity and specificity for the individual patient. The hope is that neuroimaging measures of structural changes will establish itself as means to reveal the effectiveness of rehabilitation interventions in addition to, and beyond clinical outcome measures. Although routine clinical outcome measures are utmost important to value the outcome of rehabilitation interventions, they are limited to disclose, if the trainings where effective enough to sufficiently engage the CNS to allow for any improved outcome.

## Conflict of Interest Statement

The Review Editor Nikhil Sharma declares that, despite being affiliated to the same institution as author Patrick Freund, the review process was handled objectively and no conflict of interest exists. There is a commercial interest of two authors (Kiper and Eng) relevant to the subject of the manuscript. The authors declare that the research was conducted in the absence of any commercial or financial relationships that could be construed as a potential conflict of interest.

## Footnotes

^1^www.stata.com
